# Dental Nutrition

**Published:** 1885-11

**Authors:** J. R. Walker

**Affiliations:** New Orleans


### DENTAL NUTRITION.

BY J. R. WALKER, D. D. S., NEW ORLEANS.

There are many different ways of arriving at the same conclusion. I was very much interested in the ideas expressed in several of the papers and discussions at the Minneapolis meeting, some of which were corroborative of observations which I have been making during a number of years, others differing widely from the conclusions at which I have arrived.

The general diet of residents of New Orleans, with the cistern water, used almost exclusively for potable purposes, constituting an environment differing considerably from that of any other portion of the country, and resulting in a greater amount and degree of softened, decalcified teeth than are to be found elsewhere, led me, many years ago, to a careful study of these conditions.

The rapid and extreme decalcification which I was able to observe in the mouths of those immigrating from sections of country where nature offers a more abundant supply of calcareous elements, and the recalcification occurring in the same teeth when visiting, for some time, their former homes, were to me exceedingly interesting facts.

The rapid changes which I thus saw taking place in tooth-substances proved to my mind, beyond a doubt, the existence of nutrient organs permeating the entire substance of the tooth. Not having the time nor the facilities to follow out the lead of this suggestion with the microscope, I could only call the attention of investigators to this as one of the most interesting fields for microscopic research, believing that they would find, in actual existence, that which analogy had taught me must of necessity be there.

Many excellent friends of mine took opposite views. Living in regions where such changes as I had seen were never found, they could scarcely believe them possible, and upheld the idea, with considerable tenacity, that a tooth, once formed, was a permanent institution, and liable to very little, if any, constitutional change thereafter.

Some, indeed, admitted a certain degree of decalcification, or "retrograde metamorphosis," and also the possibility of secondary deposits of lime-salts, producing pulp-nodules, or such as would drive the pulp back and protect it from approaching decay, and also the enlargement of the roots by external deposits. But they could not conceive of such a thing as a system of nutrient circulation throughout the body of the tooth, by which the lime-salts, held in solution in the blood, could be conveyed to all portions of the tooth-structure, hardening teeth that had become decalcified by fresh deposits of calcific elements, or, in other words, that the function of nutrition, with its double action of eliminating effete matters and supplying the place with new material, was in constant, active operation throughout the entire substance of the tooth, and that lime-salts could be so administered, designedly, as to supply such deficiencies in tooth substance through this nutrient circulation. Or, if they admitted the existence of any such system primarily, they insisted upon its entire obliteration at maturity, the tooth being thereafter subject to no further constitutional changes. And yet, the facts almost daily witnessed in my practice proved to my mind that there must be such a nutrient system. The results were there before my eyes, results which could be accounted for by no other theory.

Where teeth were rapidly decalcified, there must be a means of carrying off the waste material ; where teeth were hardened and recalcified by proper diet and proper treatment, necessarily there must be a means of bringing to all parts of the tooth the necessary elements for recalcification.

What my reason told me must be the case, the microscope has since shown to be the fact. From the *tubuli* of McQuillen, the *fibrillæ* of Cutler, down through all the more recent discoveries of Bodecker and others, the facts have forced a continual change of position ; the ground has been yielded little by little, until the enamel was the only part left in which the circulatory system had not been demonstrated. And this year comes no less an authority than Prof. Frank Abbott, who, in a paper read at Minneapolis, describes "the distribution of living matter in tooth-substance, even in the interstices separating the subdivisions of the enamel rods, thus raising the enamel itself to the dignity of living tissue instead of a mere mass of calcareous elements;" and establishing completely the claim that I had put forth many years ago, that the whole tooth was living matter, and subject to continual nutrient action.

I am daily seeing in my practice the direct beneficial results of the recognition of this fact of continued nutrient action, in the benefit derived from proper regulation of diet and the administration of lime-salts, in the form of *aqua calcis*, in hardening softened, decalcified teeth.

Teeth that come to me so soft and "chalky," so deficient in calcic elements that they are only fit for temporary operations before submission to constitutional treatment, are, when my instructions are followed, so hardened in the course of a few months that they are ready for permanent gold fillings. Not by the administration of "earthy phosphates," however. I failed, as utterly as did my friends Barrett, Abbott, and others, in obtaining any satisfactory results from the administration of the phosphates.

I wandered in the dark a long time, trying to restore the lacking phosphate of lime by the direct administration of the phosphates themselves. As Dr. Barrett said in the discussion of his paper read at Minneapolis, " the system will not take the elements from any ready-made source. It must elaborate its own pabulum."

But it is nevertheless true that the solution of lime faithfully administered—simple, plain lime-water—will be so decomposed and reorganized within the system as to furnish the elements necessary to harden and reconstruct poor, soft, chalky teeth.

An experience of more than fifteen years in this direction has never failed to produce the desired results, where instructions were followed, and this in hundreds of cases.

Proper diet is a valuable adjunct in this work, but diet alone, in these extreme cases, even the most faithful adherence to whole-wheat flour, oatmeal, etc., will not produce the results sought—i. e., arrest decalcification in time to save the teeth—without the accompanying use of lime-water.

It is next to impossible for people in ordinary situations to obtain the essentials of a perfectly healthy diet. I am glad to see, however, that the fashion is changing in this respect, and that oatmeal, brown bread, etc., are more readily obtained than formerly; but for those who cannot or will not adopt such diet, it is possible, by the administration of lime-water, to harden and save the class of teeth mentioned, and even to overcome hereditary tendencies in this direction, giving children good teeth, even under these unfavorable local surroundings.

It has become somewhat the fashion for the opponents of health-diet to fall back upon the *ipse dixit* that all human aliment must, of necessity, have passed through either the vegetable or the animal kingdom, or both. A little practical observation on the part of those visiting different sections of the country will prove the fallacy of this position. What makes the difference between the tall, angular, bony people—the prevailing type in the mountain regions of Tennessee, Kentucky, Virginia, the Carolinas, and of south-west Texas, where carbonate of lime abounds, and of north-west Texas, where gypsum is the prevailing formation—and the stout, round, corpulent people of the coast regions? A supposed difference in functional activity will not account for it; the lowlanders appropriate as much material and acquire as much avoirdupois as do the mountaineers. The same circulatory current that removes the calcic element, producing decalcification, would bring back new supplies and recalcify and maintain the proper integrity of the tissues, if the requisite elements were there in proper proportions.

The difference is the result, not so much in the elements of the solid food they eat, as of the water they drink.

In these days of railroads and of commercial activity in the transportation of food elements, a great uniformity prevails in the solid foods of these different sections; they nearly all use the same kind of flour for bread, the same kind of beef, and so of nearly all other solids, but the water for potable uses differs widely in the different localities. In the one case it carries lime, the elements in solution of which build up those fine osseous systems, and develop solid teeth. In the other case, the rain water used is simply a solvent, and does not furnish any mineral supplies.

The fact that the osseous system depends so largely for its supply of pabulum upon the mineral elements held in solution in the water used for potable purposes, is so apparent that the character of the teeth in a given locality can be predicted from the underlying rocks and the resultant water supply, as unfailingly as the flora and the general fauna.

Another favorite assertion is that, no matter what diet may be used, the very poorest article is rich enough in lime-salts to supply all the demands of the tissues.

Now, we know that in every one hundred parts of oxygen inhaled, an excess of ninety-six parts must be supplied in order that the four parts necessary to sustain life may be utilized; that chloride of sodium, common salt, is one of the necessities of the system, though it is eliminated unchanged; that there is such a constant elimination of waste products that all the other elements of the human tissues must be supplied largely in excess of the amount actually appropriated, yet gentlemen would restrict the supply of the calcific elements to the exact quantity found in the dried tissues by actual weight, with no allowance for the proportionate excess admitted for all other elements. Is it not more probable that nutrient action and consequent change of elements is much more rapid, consequently necessitating larger supplies than the physiological theorists have hitherto admitted, and the continual elimination of lime-salts from the system proof of this, rather than of excess of supply in the pabulum?

Those who claim that lime-salts should not be administered because they are continually eliminated, might, with as much reason, say we should breathe less, as only four parts of the one hundred of oxygen we now inhale are utilized, and ninety-six rejected as useless.

Much remodelling of many of our theories is, and will be necessary, to adapt them to facts and conditions, before we can lay down physiological axioms and say that we have the exact truth as it is in nature.

